# Recent Development in Novel Lithium-Sulfur Nanofiber Separators: A Review of the Latest Fabrication and Performance Optimizations

**DOI:** 10.3390/membranes13020183

**Published:** 2023-02-02

**Authors:** Andrew Kim, Jatis Kumar Dash, Rajkumar Patel

**Affiliations:** 1 Department of Chemical Engineering, The Cooper Union for the Advancement of Science and Art, New York, NY 10003, USA; 2 Department of Physics, SRM University-AP, Amaravati 522502, India; 3 Energy and Environmental Science and Engineering (EESE), Integrated Science and Engineering Division (ISED), Underwood International College, Yonsei University, 85 Songdogwahak-ro, Yeonsu-gu, Incheon 21983, Republic of Korea

**Keywords:** lithium-sulfur batteries, separator, carbon nanofiber, metal organic framework

## Abstract

Lithium-Sulfur batteries (LSBs) are one of the most promising next-generation batteries to replace Li-ion batteries that power everything from small portable devices to large electric vehicles. LSBs boast a nearly five times higher theoretical capacity than Li-ion batteries due to sulfur’s high theoretical capacity, and LSBs use abundant sulfur instead of rare metals as their cathodes. In order to make LSBs commercially viable, an LSB’s separator must permit fast Li-ion diffusion while suppressing the migration of soluble lithium polysulfides (LiPSs). Polyolefin separators (commonly used in Li-ion batteries) fail to block LiPSs, have low thermal stability, poor mechanical strength, and weak electrolyte affinity. Novel nanofiber (NF) separators address the aforementioned shortcomings of polyolefin separators with intrinsically superior properties. Moreover, NF separators can easily be produced in large volumes, fine-tuned via facile electrospinning techniques, and modified with various additives. This review discusses the design principles and performance of LSBs with exemplary NF separators. The benefits of using various polymers and the effects of different polymer modifications are analyzed. We also discuss the conversion of polymer NFs into carbon NFs (CNFs) and their effects on rate capability and thermal stability. Finally, common and promising modifiers for NF separators, including carbon, metal oxide, and metal-organic framework (MOF), are examined. We highlight the underlying properties of the composite NF separators that enhance the capacity, cyclability, and resilience of LSBs.

## 1. Introduction

The rise in energy-hungry electric vehicles, powerful portable devices, and large-scale renewable energy storage has increased the demand for energy-dense batteries with excellent mechanical and thermal stabilities. As Li-ion batteries are getting closer and closer to their theoretical limits [1], it is clear that next-generation batteries are required to advance civilization toward a new era of portable technologies and clean energy. Long-range electric vehicles need energy-dense and fast-charging batteries that can operate under a wide range of temperatures, physical stresses, and power demands [2]. Powerful portable devices, such as laptops, phones, and internet-of-things (IoT) technology, demand lightweight batteries with high energy capacities and minimal self-discharging [3,4]. As we generate more energy from renewable sources, such as solar [5], hydroelectric [6], thermoelectric [7], and microbial [8], we require high capacity that can store excess energy in low demand and release the stored energy on demand. Thus, there is growing interest in next-generation energy storage technologies like supercapacitors [9], sodium-ion batteries [10], and solid-state batteries [11].

One of the most promising next-generation batteries is the lithium-sulfur battery (LSB) because of its high theoretical capacity, natural abundance, and safety. LSBs have theoretical energy densities that are five times higher than Li-ion batteries, boasting a theoretical energy density of 2500 Wh kg^−1^ and a specific capacity of 1672 mAh g^−1^ [12]. However, LSBs are not yet ready for commercial adoption because of their poor cyclability and rate capability, which stem from address Li polysulfide (LiPS) shuttling (discussed in Section 2), Li dendrite formation, and the low conductivity of sulfur [13,14]. To address the shuttle effect and dendrite formation, conductive, polar, or catalytic materials like graphene [15], semiconductor quantum dots [5], double-layered hydroxides [16], and MXenes [17] may be used as additives in LSBs. Modifying commercially mass-produced polyolefin separators, such as polypropylene (PP) and polyethylene (PE) separators, is beneficial for transitioning away from Li-ion batteries. However, the intrinsically low ionic conductivity, thermal stability, electrolyte affinity, and LiPS interactivity of polyolefin separators severely limit the potential of LSBs.

**Figure 1:**
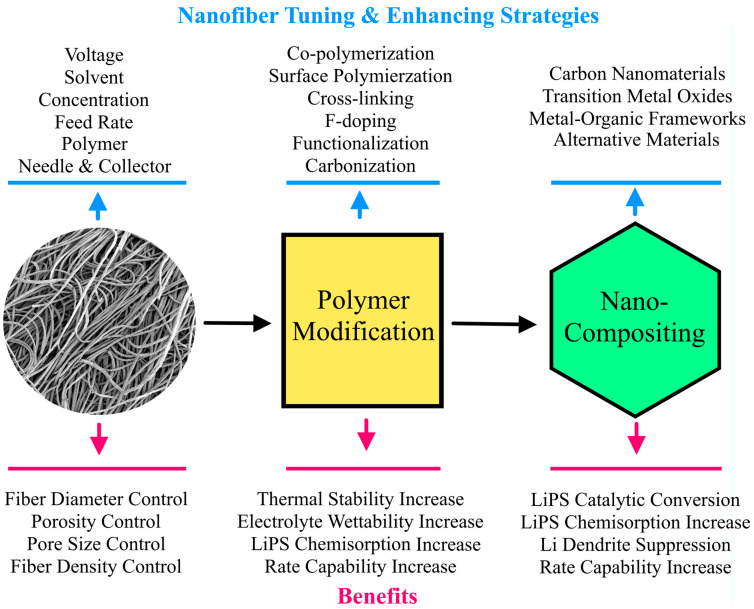
Schematic summarizing the strategies for tuning and enhancing NFs for LSBs via polymer modification and nanocompositing. The benefits of modifying NFs for LSBs are also summarized.

Nanofiber (NF) separators are highly porous, thermally stable, low cost, and easily modifiable alternatives to polyolefin separators [18,19,20]. Because NFs have high porosity and polar functional groups (in some polymer NFs), they also have higher electrolyte affinity and LiPS-adsorbing properties [21]. Hence, NFs have been used as interlayers or surface modifications to polyolefin separators in LSBs [22,23,24]. However, a recent trend has been growing for the fabrication of novel NF separators without the limiting polyolefin component. While there are already reviews on NFs for cathodes and interlayers in LSBs [25,26,27], we are unaware of any reviews focusing on NF separators in LSBs. In this review, we discuss the performance of LSBs with NF separators fabricated from various polymers. Novel CNT-based NF separators are also analyzed for their fabrication method and enhancing features. Various NF composite separators have also been investigated to improve the suppression of the shuttle effect and Li dendrite growth. Herein, we focus on the most tested additives, including carbon, transition metal oxide, metal-organic framework (MOF), and other promising nanomaterials. Figure 1 summarizes the benefits of fine-tuning and enhancing NF separators for LSBs via polymer modifications and nanocompositing. We hope this review inspires more work into developing NF separators to make LSBs commercially viable and reach the theoretical limit of LSBs.

## 2. Lithium Polysulfide Shuttle Effect

**Figure 2:**
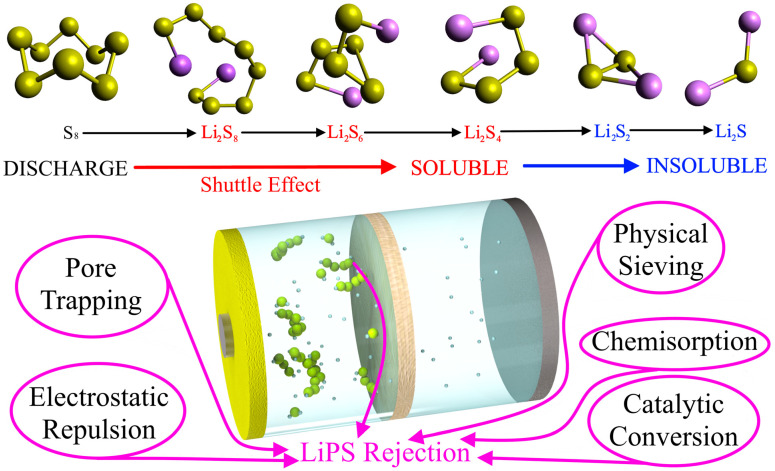
Schematic diagram showing the conversion of S_8_ to Li_2_S with intermediate LiPSs during LSB discharge (Top). The insolubility/solubility of the LiPSs is noted by red/blue colors. The main LiPS rejection mechanisms employed by NF separators.

One of the biggest hindrances to commercializing LSBs is the shuttle effect, which decreases the amount of usable sulfur and Li, decreases ionic conductivity, and severely limits battery cyclability. Herein, we briefly overview the shuttle effect from the perspective of LSB discharge. Details on the redox process of LiPS molecules have been provided elsewhere [28,29,30]. Figure 2 illustrates a typical discharge process in which S_8_ is converted to Li_2_S via multiple stable intermediates of varying sulfur lengths. The long-chain polysulfides (Li_2_S_8_, Li_2_S_6_, and Li_2_S_4_) are soluble in common LSB electrolytes, so they are driven from the sulfur cathode to the Li anode via diffusive and electromotive forces. The long-chain polysulfides at the anode are then semi-permanently reduced into insoluble Li_2_S. The consequences are (a) reduced active material, which decreases capacity; (b) decreased Li-ion diffusion due to a build-up of non-reactive LiPSs; (c) large volume expansion that may break the enclosed LSB; and (d) fast self-discharging that makes commercialization difficult. While using solid-state electrolytes can effectively prevent the shuttle effect, LSBs with even the latest solid-state electrolytes are not commercially viable due to poor ionic conductivity and slow redox kinetics that limit energy storage and power density [31,32,33]. Thus, there have been great efforts to mitigate the shuttle effect using novel NF separators in liquid electrolytes.

NF separators mainly rely on five mechanisms for LiPS rejection: pore trapping, electrostatic repulsion, physical sieving, chemisorption, and fast catalytic conversion. The nanoporous structure is conducive for effective pore trapping and ionic sieving, especially for long-chain polysulfides. Moreover, NF separators can use materials with a negative surface charge for electrostatic repulsion. Novel NF separators also have functional groups or embedded nanoparticles that provide abundant active sites for chemisorption and fast catalytic conversion of soluble long-chain LiPSs into insoluble short-chain LiPSs. The specific strategies for enhancing LiPS rejection mechanisms are highlighted in the rest of this review.

## 3. The Electrospinning Technique

The fundamentals of electrospinning are briefly discussed because it is the primary method of fabricating novel NF separators for LSBs because of its low cost, simple setup, and easy tunability. Compared to other spinning methods, they yield fibers that have nanometer-scale diameters, controllable nanostructures, consistent porosity, high surface-to-volume ratios, and stable nanocomposite structures [34,35]. A detailed discussion on the history, setup, and analyses of the electrospinning technique and its variations can be found in reviews by Bhardwaj et al. [34], Teo et al. [36], Ahmed et al. [37], and Li et al. [38]. Information on other non-electrostatic methods for NF fabrication, such as melt-blowing, wet-laying, flash-spinning, and other mechanical spinning techniques, can be found in reviews by Song et al. [39] and Nayak et al. [40]. However, such methods are not commonly employed for LSB separators because they are not as facile, familiar, or easy to manipulate. Newer and more unique techniques exist, such as foam-based needleless electrospinning and two-level coil edge electrospinning [39], but the principle behind standard electrospinning is discussed because it is the most commonly used method for LSB separators.

**Figure 3:**
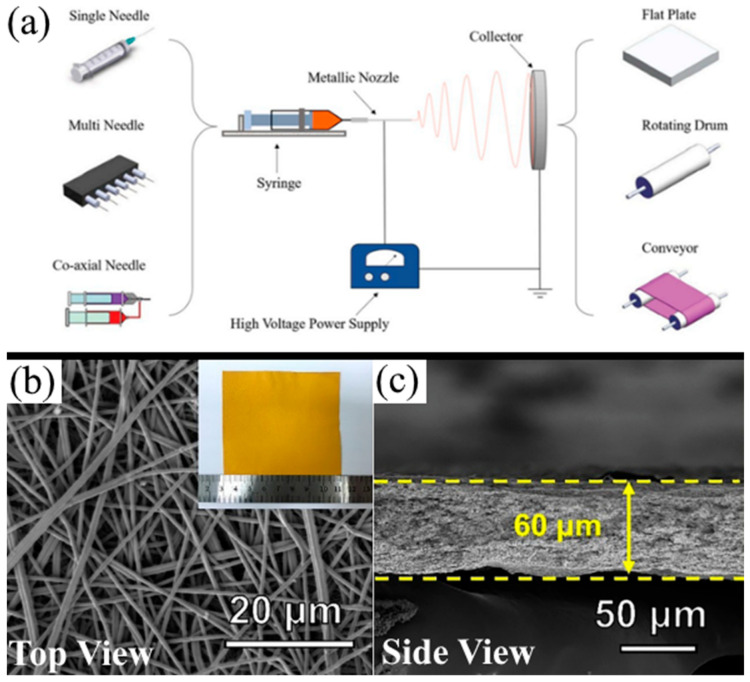
(**a**) A schematic diagram of a standard electrospinning process. Reprinted/adapted with permission from Ref. [35]. (Copyright 2019, Elsevier). SEM (**b**) top view and (**c**) side view of an ammoniated PAN NF separator. A digital photograph of the NF is provided in the insert in (**b**). Reprinted/adapted with permission from Ref. [41]. (Copyright 2020, Elsevier).

Figure 3a shows a typical electrospinning setup with variations in the type of syringe needle and collector plate. A polymer solution is placed into a syringe (spinneret) that has a thin, electrically charged needle at the end. A high-voltage power supply raises the voltage at the tip of the needle to a critical voltage (typically higher than 5 kV). Due to the large electric field, the solution becomes charged, and when the repulsive forces in the solution overcome surface tension, the polymer solution is ejected as thin strands. These strands stretch and bend as the solvent evaporates. The charged polymer is electrically neutralized when it comes into contact with an electrically grounded collector. Figure 3b,c shows the top and side of the fiber morphology of an electrospun ammoniated PAN NF [41], which is the typical morphology of the NF separators discussed in this review. The polymer solution, needle, collector, working voltage, and feed rate can be adjusted to yield NFs with highly tunable diameters, lengths, porosity, surface charge, and density. Many investigations discussed in this review do not explicitly optimize electrospinning parameters; therefore, their results regarding LiPS rejection, Li-ion conductivity, and electrolyte wettability may be suboptimal. This is hopeful news because such studies leave room for fine-tuning to yield commercially competitive novel separators for LSBs.

## 4. Novel Nanofiber Separators

### 4.1. Polymer-Based Nanofibers

**Figure 4:**
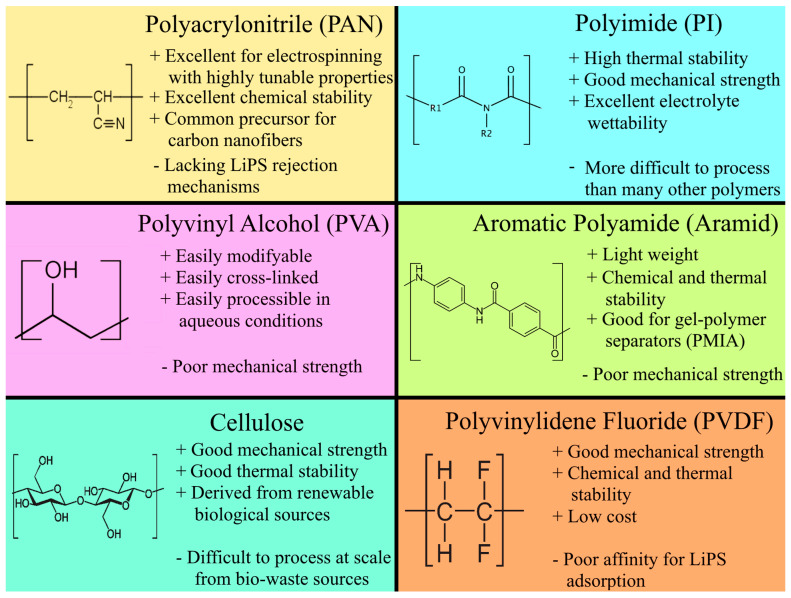
The advantages and disadvantages of promising polymer materials for NF separator fabrication and application in LSBs.

Polymer-based NFs are the most popular novel separators for LSBs, owing to their almost limitless customizability, facile fabrication, high thermal resistance, light weight, and mature scale-up technologies. The performance of LSBs with exemplary polymer-based NF separators is summarized in Table 1. The chemical monomers, advantages, and disadvantages of the various polymer NF separators for LSBs are summarized in Figure 4. While NF separators are generally thicker than polyolefin separators, NF separators have much greater porosity than polyolefin separators, resulting in significantly higher ionic conductivity despite the higher separator thickness. The greater separator thickness also slightly increases the battery volume. However, the higher ionic conductivity and electrolyte wettability can increase the energy density to greatly outweigh the slightly larger volume.

One of the most commonly electrospun polymers for LSB separators is polyacrylonitrile (PAN) because it is easy to process, resistant to oxidation, and has high thermal resilience [42,43,44]. PAN is also a common precursor for CNFs [45], which are examined in more detail in Section 3. Many studies have investigated coating commercial separators with PAN NFs as excellent interlayers that improve electrolyte wettability, thermal stability, Li-ion conductivity, and polysulfide rejection in Li-ion batteries and LSBs [46,47,48,49,50]. However, using only PAN NFs as the separator results in far greater thermal stability, significantly improving battery safety than using PAN NFs as modifiers for existing polyolefin separators [51]. While it is possible to use unmodified PAN NFs in Li-ion batteries, the high porosity and lack of LiPS adsorption sites leave LSBs vulnerable to the shuttle effect. Conductive and electrocatalytic nanoparticles like carbon nanoparticles [52] or Al_2_O_3_ [53] may be composited with PAN NFs to improve LiPS adsorption and catalytic conversion. Composite NF separators are discussed in more detail in Section 4.

PAN NF separators may be modified with other polymers or adsorptive groups. For example, Hu et al. [41] ammoniated PAN (APAN) NFs with polyethyleneimine (PEI) via chemical grafting to introduce various amino groups to the NFs. Compared to PP and unmodified PAN separators, the amino groups in the APAN separator helped form a stable electrolyte interface (SEI) layer, which decreased electrolyte consumption and regulated Li dendrite deposition. The amino groups also improved electrolyte wettability and LiPS adsorption. The modified PAN/PEI NF structure also physically blocked LiPS shuttling with its branched structure. An LSB with the APAN separator had 12% and 30% higher initial capacity and 38% and 42% higher capacity retention than an LSB with PAN and PP separators, respectively. Zhu et al. [54] modified a PAN/poly(acrylic acid) (PAN/PAA) with abundant carboxyl functional groups with an ethanol vapor treatment. This endowed the NF separator with a highly negative surface charge that repelled LiPS and improved electrolyte wettability.

**Figure 5:**
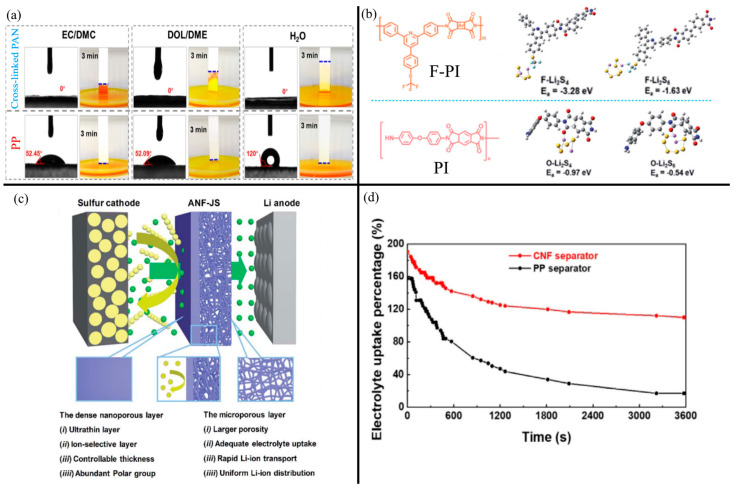
(**a**) Electrolyte wettability and meniscus behavior of common LSB electrolytes and water on cross-linked PAN NF separator and PP separator. Reprinted/adapted with permission from Ref. [55]. (Copyright 2021, American Chemical Society). (**b**) Chemical structures of F-PI and PI and their binding energies to Li_2_S_4_ and Li_2_S_6_. Reprinted/adapted with permission from Ref. [56]. (Copyright 2020, Royal Society of Chemistry). (**c**) Schematic of a Janus-type aramid NF separator (ANF-JS), with the advantages of the dense and microporous structures listed. Reprinted/adapted with permission from Ref. [58]. (Copyright 2022, Royal Society of Chemistry). (**d**) Comparison of electrolyte uptake and storage time between a standard PP separator and CNF separator. Reprinted/adapted with permission from Ref. [59]. (Copyright 2022, Elsevier).

Cross-linking PAN NFs with other polymers can improve the mechanical properties and fine-tune porosity. Recently, Hu et al. [55] cross-linked PAN NFs with amphiphilic poly(ethylene glycol) diacrylate (PEGDA)-grafted siloxane (TPT). The cross-linked PAN NF separator had a high 77.9% porosity, almost 60% higher than a standard PP separator. The polar oxides (Si−O−Si, C=O, C−O, and C=N) from the TPT improved electrolyte wettability, as shown by the lower contact angle and greater capillary action in Figure 5a. Compared to unmodified PAN, the cross-linked PAN NF separator exhibited a six-times-higher tensile strength of 18.8 MPa and a 164-times-higher Young’s modulus, making the modified PAN NF separator viable for stand-alone use. The strong cross-linking also reduced thermal shrinkage, with no noticeable shrinkage at 160 °C, whereas PP separators are fully melted by 160 °C. The polar oxide groups also improved LiPS adsorption, resulting in a 78% capacity retention after 300 cycles.

Various other polymer NFs have been used as LSB separators. Polyimide-based NFs are promising for their excellent thermal stability and mechanical strength that promote safety and various polar groups that improve electrolyte affinity and LiPS adsorption. Luo et al. improved the thermal safety of PI NF separators via fluorination [56]. While both un-fluorinated PI and fluorinated PI (F-PI) showed no sign of thermal shrinkage even at 140 °C, the F-PI separator was almost mostly non-flammable and had a much shorter self-extinguishing time due to the -CF_3_ groups while the pristine PI NF was quickly combusted. Moreover, the fluorine groups in F-PI exhibited strong electrostatic repulsion against LiPS while improving Li-ion diffusion. As shown in Figure 5b, the F-PI had larger adsorption energies due to the strong interactions between F and Li_2_S_4_/Li_2_S_6_ than the oxygen groups with the LiPS in unmodified PI. Thus, the LSB with an F-PI NF separator exhibited a very high long-term capacity retention of 95.6% after 500 cycles at 1 C. In a later study, Luo et al. [57] tuned the pore sizes and electrochemical properties of a PI NF separator by modifying it with a top layer of polyamide/polyvinyl alcohol (PA/PVA) via interfacial polymerization (IP). Like the aforementioned fluorination, the PA/PVA possessed a highly negative charge, enabling the modified PI separator to repel LiPS while improving Li-ion diffusion. The IP process created a nanoporous structure, decreasing the porosity from 92.1% for pristine PI NFs to 75.1% for PA/PVA-modified NFs, resulting in more LiPS rejection and sufficiently high electrolyte uptake for excellent Li-ion mobility.

Polyvinyl alcohol is a water-soluble and non-toxic polymer with higher thermal stability than commercial polyolefin separators and can be easily modified due to its hydroxyl groups [60]. Hence, it is common to fabricate multifunctional PVA-based separators by combining various functional groups and nanoparticles [61,62,63]. Cross-linking is also common for PVA-based separators [64,65,66] to improve mechanical strength and control pore sizes. For example, Zhou et al. [67] recently fabricated a cross-linked PVA/poly (lithium acrylic acid) (PVA/PAA-Li) NF separator via electrospinning followed by heat treatment. Cross-linking decreased porosity and improved tensile strength as expected, and the highly polar PVA/PAA-Li enabled a high electrolyte uptake of 430% (two times higher than a Celgard separator).

Aromatic polyamides, frequently called aramids, are often electrospun from Kevlar into nanofibers that boast excellent mechanical and thermal stability [68]. Many aramid NF (ANF) separators have been designed as alternatives to polyolefin separators in Li-ion batteries [20,69,70,71,72] and only recently in LSBs [58]. Liu et al. [73] electrospun a poly(m-phenylene isophthalamide) (PMIA) solution to yield a 3D network of 90 nm fibers. Compared to a standard PP separator, the ANF separator exhibited better electrolyte wettability, uptake, and retention in addition to thermal stability even at 200 °C and self-extinguishing capability. These advantages were afforded by the porous nanofiber structure and chemically/thermally resistant PMIA material properties. Moreover, the LSB with the aramid NF separator exhibited a 38% higher initial capacity than with a standard PP separator. Moreover, aramid NFs can be fabricated as sol-gel-type separators [58]. For example, Pei et al. [58] fabricated a Janus-type separator with a highly porous side facing the Li anode and a dense nanofiltering side facing the sulfur cathode. Creating a sol-gel film of ANFs was critical because the dense film was synthesized via a dry-wet phase inversion process. The Janus-type structure enabled high ionic conductivity while having superior LiPS rejection, indicated by a low 0.019% capacity decay per cycle at 1 C. The advantages of the Janus structure with a dense and loose side are summarized in Figure 5c.

Poly-m-phenyleneisophthalamide (PMIA) is a specific type of aramid polymer that is promising for gel-polymer electrolyte NF separators in LSBs. Unlike in traditional configurations where the electrolyte and separator are separate materials, gel-polymer electrolyte separators have electrolyte impregnated and immobilized within the separator matrix [74,75]. Thus, LSBs with gel-polymer electrolyte separators have reduced flammability than batteries with liquid electrolytes and better ionic conductivity than solid electrolytes [76]. While poly(ethylene oxide) has also been used to make gel-polymer electrolyte separators in LSBs [77], the latest work in gel-polymer electrolytes involves fabricating gel-polymer NF separators with PMIA. Still, PMIA NF separators must be modified to improve mechanical stability, electrolyte affinity, and LiPS adsorption before they may be commercially viable. One method is to modify PMIA NFs with a fluorinated emulsion. Xiang et al. [78] found that fluorination decreased the electrolyte contact angle from 66⁰ to 23⁰ and increased electrolyte uptake by 12%. The highly polar fluorine groups also reduced pore size by 18% but increased pore volume by 60%, resulting in improved LiPS rejection while maintaining high Li-ion diffusion. The electrolyte affinity and LiPS rejection was further improved by co-doping PMIA with 3, 4-ethylene dioxyethiophene (EDOT). The S and O atoms in EDOT increased the polarity of the NF separator for improved electrolyte absorption and interacted strongly with LiPSs for reduced shuttling. The fluorinated emulsion also improved the mechanical strength of the NF separator by 19%, and EDOT modification improved strength by 41%. This was due to decreased NF diameters with an increased density of fiber roots. Similar benefits of using a fluorinated emulsion were reported in recent investigations by Zhao et al. [79], Yang et al. [80], and Deng et al. [81,82]. In all aforementioned studies, fusing a fluorinated emulsion is not enough, and other dopants such as nanoclays [83], transition metal oxides [81], polar biomolecules [80], and catalytic compounds should be added [80]. Such composites are categorized and discussed in more detail in Section 4.

Cellulose has recently gained attention as a suitable modifier or replacement for standard polyolefin separators due to its excellent electrophilic properties, strong anisotropic structure, and low cost, amongst other features. While cellulose has been used in various components in LSBs, as summarized in a recent review by Zhang et al. [84], their fabrication into NFs and application in LSBs is only recently gaining attention. Li et al. [59] fabricated from a wood slurry via a wet processing method in an isopropanol solution. The ratio of water to isopropanol was important because high isopropanol concentrations yielded larger pore sizes, with the ideal ratio at 95 vol% isopropanol. The high 98% porosity and polarity resulted in significantly higher electrolyte uptake, retention, and wettability, and ionic conductivity than a PP separator (Figure 5d). Despite the high porosity, the cellulose NFs had a 60% reduction in pore size than PP, resulting in improved LiPS rejection. Instead of plant-derived cellulose, Li et al. [85] fabricated bacterial cellulose (BC) NFs and further enhanced the BCNFs via oxidation. The formation of abundant hydroxyl and other polar oxygen groups on the BC chains improved Li-ion conductivity by 57% than a PP separator. Moreover, the high polarity of the oxidized BCNFs regularized Li stripping/plating, reducing the formation of Li dendrites. The abundant polar groups also exhibited strong adsorption for LiPS. The naturally thermal resistant properties of cellulose led the BCNF separator to withstand extremely high temperatures up to 350 °C before melting and decomposition.

Polyvinylidene fluoride (PVDF) is also a common polymer that is electrospun due to its excellent mechanical, chemical, and thermal stability in addition to its non-toxicity, flexibility, and low cost [86,87]. Zhu et al. [88] found that the PVDF NF had a 50% higher porosity and almost two-times-higher electrolyte uptake than a standard PP separator. The higher porosity and electrolyte uptake allowed faster Li-ion diffusion, resulting in improved rate capability, indicated by an 18% higher initial capacity at 0.5 C but a 56% higher specific capacity at a higher current density of 2 C. However, an LSB with a PVDF separator had similar long-term cycle stability as a PP separator due to its poor affinity for LiPS. Thus, PVDF NF separators need to be modified with polar materials before PVDF NFs can be used in LSBs, which are discussed in detail in Section 4. However, it is possible to improve LiPS rejection by fabricating a PVDF-based copolymer NF. Shi et al. [89] electrospun a PVDF/polymethylmethacrylate (PMMA) copolymer. The ester groups in the PMMA had higher binding energies for the LiPSs, resulting in improved rejection rates. This was indicated by a small 0.095% decay per cycle in specific capacity with a PVDF/PMMA separator compared to a 0.185% decay per cycle at 0.2 C.

While polymer-based NF separators are non-conductive to prevent electrical short-circuiting, it is advantageous to have a conductive layer facing the sulfur cathode to improve the rate capability of the LSB and increase LiPS conversion kinetics. A Janus-type separator with conductive polymer NFs on top of an electrically insulative NF separator was fabricated by coating a non-conductive polyvinylpyrrolidone NF with conductive polypyrrole (PPy) via vapor-phase polymerization [90]. The improved conductivity of the LiPS rejection layer improved the rate capability, with around a 45%-higher capacity at a high current density of 1 A g^−1^. Moreover, the rapid reduction of LiPS, enabled by fast electron transfer, enabled higher sulfur utilization for an 11% higher initial capacity than with a single-layer NF separator.

### 4.2. Carbon-Based Nanofibers

CNFs are 1D structures with a diameter of around 100 nm and lengths of 200 μm and are frequently fabricated via the carbonization of polymer nanofibers like PAN [91,92] (discussed in Section 4.1). While CNFs have been widely used as modifiers to polyolefin separators or as interlayers in LSBs [93,94,95,96,97,98,99], there has been growing interest in implementing CNFs directly as separators. However, CNF separators cannot be used directly as separators in LSBs, owing to their poor LiPS-rejection capabilities. Moreover, CNFs must be modified with a layer of insulating material to prevent short-circuiting. Herein, we discuss designing non-polyolefin separators based on CNFs for LSBs.

Before discussing CNF separator modifications, it is crucial to differentiate CNFs from CNTs. Even though CNFs are structurally quite different from CNTs, they are frequently and wrongly used interchangeably in the scientific literature because both are thin, carbon-based, 1D strands that can yield a highly conductive and porous structure. For example, in the paper by Baik et al. [100], they wrote “CNT” in the title of the article but used “CNF” in the body of the work. The main differences between CNTs and CNFs are in the geometries of their 1D structures. CNTs are more tubular and akin to rolled-up graphene sheets with atomic-level defects, such as lattice dislocations [101]. In contrast, CNFs consist of conical or planar layers stacked together to yield a fiber [102]. Due to the stacking structure, CNFs are more semiconducting than CNTs and have more chemically active sites, allowing them to be more useful as catalysts [92]. This work discusses CNFs-based separators because there are many reviews on CNT-based separators [103,104,105,106,107], but we are unaware of any reviews covering novel CNF separators specifically for LSBs.

One of the most common methods of fabricating CNFs is via the carbonization of polymer NFs created from electrospinning. For example, Wang et al. [22] fabricated CNFs by carbonizing a PVP/polytetrafluoroethylene electrospinning solution at 1000 °C under a N_2_ atmosphere. It is easy to take advantage of the electrospinning process by adding nanoparticles to the electrospinning solution to yield CNFs embedded with the nanoparticles. Xu et al. [108] similarly carbonized PAN NFs to yield CNFs. An electrically insulating layer of PAN NFs was then directly electrospun onto the CNFs, to yield a bilayer. The bilayer is essential for separators with highly conductive layers to prevent short-circuiting. Compared to PP and PAN NF monolayer separators, the CNF/PAN NF separator had a 40%-higher capacity retention, owing to improved LiPS conversion with higher electron mobility. The rate capability of the bilayer separator also improved with a capacity of 900 mAh g^−1^ with CNF/PAN NF separator at 2 C but only 280 and 100 mAh g^−1^ with PAN NF and PP separators, respectively. However, the LiPS rejection was still poor, with only 43% retention after 300 cycles at 0.5 C. Zhang et al. [45] similarly found a poor 37% capacity retention for a CNF/PAN NF separator after 200 cycles at 0.2 C. This is because CNFs, such as their precursor polymer NFs, often lack polar functional groups with high affinity for LiPSs.

**Figure 6:**
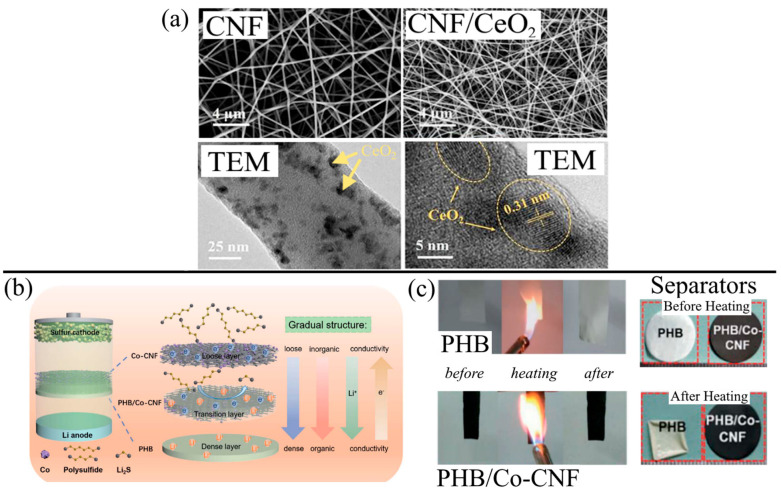
(**a**) SEMs of CNF (Top Left) and CNF/CeO_2_ (Top Right) and TEMs at low magnification (Bottom Left) and high magnification (Bottom Right). Reprinted/adapted with permission from Ref. [45]. (Copyright 2020, Elsevier). (**b**) Schematic showing the gradual changes from the loose Co-CNF layer to the dense PHB layer and the consequences of the bilayer structure. (**c**) The flame retarding ability of PHB and PHB/Co-CNF (Left) and thermal stability at 160 °C for the PHB and PHB/Co-CNF separators. Reprinted/adapted with permission from Ref. [109]. (Copyright 2022, Royal Society of Chemistry).

The modification of CNFs for improved LiPS rejection is discussed in detail in Section 4. However, the effects of nanoparticle additives on CNFs are briefly discussed. Zhang et al. [45] added CeO_2_ nanocrystals to the PAN solution and carbonized the electrospun fibers to yield CNF-CeO_2_. The addition of CeO_2_ had smaller fiber diameters due to how the CeO_2_ affected the surface tension, electrostatic repulsion, and viscoelastic forces in the electrospinning solution. The porosity was slightly lower without CeO_2_ at 72% porosity, which was much higher than PP (33% porosity) (Figure 6a). Even though electrolyte uptake decreased by 6% with the addition of CeO_2_, the CNF-CeO_2_ separator had excellent electrolyte wettability due to its excellent porosity, indicated by the 0⁰ electrolyte contact angle.

Instead of electrospinning CNFs onto an electrically insulative layer, CNFs may be attached to an insulating layer via vacuum filtration. Kong et al. [110] vacuum-filtered PAA-based CNFs through a PI NF substrate to yield a Janus-type NF separator. Similarly, Liang et al. [109] modified a Co-CNF layer with a non-conducting poly(vinylidene fluoride-co-hexafluoropropylene)/boron nitride (PHB) layer. As shown in Figure 6b, the tri-layer separator consisted of a highly porous Co-doped CNF layer facing the S cathode, a transition layer between the Co-CNF and PHB, and a dense PHB layer facing the Li anode. The Co-CNF was fabricated by carbonizing an electrospun solution of Co nanoparticles and PAN, and this layer provided multiple key functions. First, the CNFs provided flame resistance and thermal stability to the separator. As shown in Figure 6c, the PHB separator alone suffered from burning and thermal shrinkage at 160 °C, while CNFs showed no signs of flame or high-temperature damage. Secondly, the shuttling effect was greatly reduced by LiPS adsorption between Co nanoparticles and sulfur in the polysulfides and physical blocking by the nanoporous fibers. Lastly, the porous structure of the CNFs increased electrolyte wettability, capacity, and retention, improving the ionic conductivity of the LSB. While the PHB layer was a necessary insulating layer, the main role of LiPS rejection and improved thermal stability was the CNF separator.

**Table 1:** The performance and notable developments LSBs with novel polymer-based and CNF separators.

Nanofiber Material	Initial Capacity (mAh g^−1^)	Current Density (C)	# of Cycles	Capacity Decay (% per Cycle)	Highlights	Ref.
APAN/PEI	728	2	500	0.14	The ammino groups in APAN and PEI formed a thick SEI layer that regulated Li dendrite deposition	[41]
PAN/TPT	960	0.1	300	0.07	Cross-linking improved the tensile strength and Young’s modulus of the NF separator	[55]
F-PI	754	1	500	0.01	–CF_3_ groups endow excellent flame resistance	[56]
PA/PVA/PI	1380	0.2	500	0.1	Highly negatively charged PA/PVA improves LiPS rejection via Coulombic repulsion	[57]
PPTA	807	1	1000	0.02	Janus-type structure enabled high ionic conductivity with excellent LiPS rejection	[58]
PVA/PAA-Li	487	1	400	0.13	High polarity of PVA and PAA greatly improved electrolyte uptake	[67]
PMIA	1093	0.2	350	0.16	Improved thermal, mechanical stability than PP because of natural aramid properties	[73]
F-PMIA/EDOT	852	0.5	200	0.12	EDOT co-polymer increased polarity and LiPS adsorption	[78]
BC	1250	0.25	100	0.21	Oxidation of BC yielded abundant polar groups that reduced Li dendrite formation	[85]
PVDF-PMMA	950	0.2	200	0.08	The polar ester groups in PMMA enabled better LiPS rejection via chemisorption	[89]
CNF/PAN	1278	0.2	200	0.31	CNFs with CeO_2_ modifiers become thinner but retain high porosity for excellent electrolyte retention	[45]
CNF/PAN	923	0.5	300	0.22	Conductive CNFs improved rate capability and slightly restricted the shuttle effect	[108]
Co-CNF/PHB	950	0.5	150	0.27	Although there is rapid decay initially, the capacity plateaus after 20 cycles to 580 mAh g^−1^ and only decreases to 567 mAh g^−1^ after 130 more cycles.	[109]
CNF/PI	955	1 *	200	0.07	CNFs trap LiPS and provide fast electron transport for fast redox kinetics	[110]

* Current density measured in A g^−1^ instead of C rate. APAN (ammoniated polyacrylonitrile); PEI (polyethyleneimine); TPT (poly(ethylene glycol) diacrylate (PEGDA)-grafted siloxane); F-PI (fluorinated polyimide); PA/PVA/PI (polyamide/polyvinyl alcohol/polyimide); PVA/PAA-Li (polyvinyl alcohol/poly (lithium acrylic acid); PMIA (poly(m-phenylene isophthalamide)); PPTA (poly(p-phenylene terephthalamide)); BC (bacterial cellulose); F-PMIA/EDOT (fluorinated poly-m-phenyleneisophthalamide/3, 4-ethylene dioxyethiophene); PHB (poly(vinylidene fluoride-co-hexafluoropropylene)/boron nitride).

## 5. Composite Nanofiber Separators

**Figure 7:**
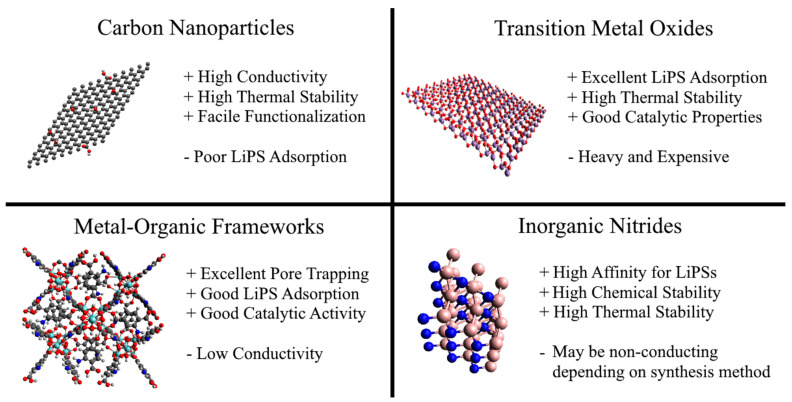
Schematic showing the basic structure of carbon nanoparticles, transition metal oxides, MOFs, and inorganic nitrides. The advantages (+) and disadvantages (-) are summarized.

While NF separators are promising alternatives to polyolefin separators due to their higher porosity, electrolyte wettability, and thermal stability, NF separators alone are insufficient for commercializing LSBs due to their inability to reject polysulfides. Some methods of improving LiPS rejection are functionalization and heteroatom doping (as discussed in Section 4), but these are often insufficient. Thus, many recent advancements in NF separators investigate compositing NFs with conductive, adsorptive, and catalytic compounds. Herein, we analyze the benefits of modifying NF separators with carbon-based nanoparticles, transition metal oxides, MOFs, and other promising materials. Figure 7 shows the basic structure of various additives and summarizes their advantages and disadvantages. The performance of LSBs with the various modified NF separators are summarized in Table 2.

### 5.1. Carbon Composites

Carbon-based materials are added to LSBs primarily to improve the redox kinetics of soluble long-chain polysulfides into insoluble short-chain polysulfides to mitigate the shuttle effect and improve the battery’s rate capability [111,112,113,114]. Carbon materials, such as graphene oxide (GO), with highly polar or electronegative functional groups can also electrostatically repel LiPS. Zero-dimensional carbon or graphene nanoparticles can be easily added to an electrospinning solution and be directly electrospun into carbon composite NFs [115]. For example, Zhu et al. [116] added graphene oxide nanoparticles (GO) into a PAN solution in a 1:10 (GO:PAN) weight ratio. The GO-modified PAN NFs had a smaller diameter of 600 nm (compared to 850 nm for PAN) and slightly higher porosity than the PAN NF separator. The GO nanoparticles endowed the NF surface with a negative charge that repelled anionic polysulfides, resulting in a 25%-higher capacity retention than with an unmodified NF separator. Moreover, the rate capability was greatly improved with the addition of conductive GO nanoparticles, indicated by a 31%- and 161%-higher capacity than PAN and PP, respectively, at a high current density of 2 C.

In a very recent study, Leng et al. [52] fabricated a novel bilayer separator based on two different NF layers. The carbon composite layer facing the sulfur cathode was fabricated by electrospinning carbon black (CB) with a two-polymer solution of PAN and poly(vinylidene fluoride-co-hexafluoropropylene) (PVDF-HFP) and VOOH nanoparticles. The resulting composite NF is referred to as CVPHP. The layer facing the anode side was fabricated by heat treating the CVPHP nanofiber at 155 °C to promote cross-linking between PAN and PVDF-HFP. The cross-linked support layer was mechanically more robust with a two-times higher tensile strength and higher thermal stability even at 250 °C. The CB in the cathode layer improved the rate capability of the LSB by improving ionic conductivity and redox kinetics, indicated by about a 50%-higher capacity retention than with a PAN NF separator at a high current density of 2 C.

Similarly, Zhang et al. [88] electrospun a reduced GO (rGO)/PVDF solution onto a PVDF NF substrate to yield a double-layer separator with two different thicknesses for the rGO/PVDF layer. Having the second layer that was porous and electrically insulative was critical to prevent short-circuiting. Increasing the thickness of the rGO/PVDF layer by 27% increased the electrolyte uptake by 7% and was 23% higher than without rGO. The improved electrolyte uptake was due to the very high surface area of the rGO nanosheets. Moreover, the charge transfer resistance of the LSB decreased by 42% with rGO, resulting in a high capacity of 590 mAh g^−1^ at a current density of 2 C (only 14 mAh g^−1^ for unmodified PVDF). Zhou et al. [117] also found improvements to the rate capability of the LSB due to rGO additives in the PAN nanofibers. Overall, modifying NF separators with carbon nanomaterials is a low-cost, facile, and effective way to improve LSB rate capability and capacity retention. However, carbon-based modifications are usually insufficient modifiers due to their poor adsorptive affinity for LiPSs and can introduce short-circuiting issues [118]. Hence, carbon-based modifications are often done as surface-level modifications on the cathode side of LSBs.

### 5.2. Transition Metal Oxide Composites

Transition metal oxides, such as SnO_2_, Co_3_O_4_, V_2_O_5_, MnO_2_, CoMoO_4_, and NiCo_2_O_4_, have been added to various components of LSBs because of their high adsorption affinity for LiPSs and their catalytic properties for LiPS conversion [119,120,121,122]. The metal oxides can be deposited onto NFs [123], grown on the NFs in situ [124], or electrospun directly with a polymeric solution [125]. Many publications on metal oxides in LSBs focus on cathode materials, LSB interlayers, or coatings for polyolefin separators. Few studies have implemented metal oxides into novel NF separators, despite the promising catalytic and adsorptive properties of metal oxides. Herein, we discuss such exemplary works.

Adding metal oxides such as CeO_2_ [45] has improved the rate capability of CNF/PAN NF separators by improving LiPS reduction kinetics, indicated by a 36%-higher capacity at a high current density of 2 C. While conductive CNFs can provide fast electron transport pathways (Section 4.2), they have low affinities for LiPS adsorption, which limit the redox rate. Thus, adding highly adsorptive CeO_2_ nanoparticles was needed to make the most out of the higher conductivity, significantly increasing the capability of the LSB. Consequently, the LSB can have higher sulfur loadings and improved sulfur utilization, resulting in an increased specific capacity. Pei et al. grew Fe_2_O_3_ nanoparticles in situ via a hydrothermal reaction [58]. While such a synthesis process would cause problems Fe_2_O_3_ crystallization in polyolefin separators that could destroy the uniform porosity, the facile hydrothermal process on the NF separators yielded highly stable and uniform nanocrystals. A simple adsorption experiment showed that the Fe_2_O_3_-modified separator could decolorize an opaque yellow LiPS solution to a more colorless and transparent state. Thus, the separator with Fe_2_O_3_ had around a 20%-higher specific capacity after 100 cycles at 1 C.

Guo et al. [53] fabricated a PAN/Al_2_O_3_ NF by directly electrospinning a solution of PAN and Al_2_O_3_ nanoparticles. The Al_2_O_3_ nanoclusters were anchored on the surface of the PAN NFs, increasing the number of active sites available for LiPS adsorption. The Al_2_O_3_ also improved the electrolyte wettability, with a 57% decrease in electrolyte contact angle. This was due to the strong interactions between the metal ions in the metal oxides and anions in the electrolyte. Moreover, the metal oxides improved the chemisorption of LiPS, resulting in a 49% increase in capacity retention after 100 cycles at a current density of 200 mA g^−1^.

Metal oxides in the Li anode-facing side can also improve LSB longevity by decreasing Li dendrite formation. Wu et al. [126] coated the anode side of a PAN NF separator with Li_6.4_La_3_Zr_1.4_Ta_0.6_O_12_ (LLZTO) via magnetron sputtering. The higher surface area of the NFs and polarity of LLZTO increased electrolyte affinity. Moreover, the conductive LLZTO decreased the bulk resistance of the LSB by 43% and increased ionic conductivity by 43%. The conductive pathways provided by LLZTO on the anode side encouraged the uniform deposition of Li-ions on the anode, decreasing dendrite formation.

### 5.3. Metal-Organic Framework Composites

MOFs are one of the most promising additives to LSB separators, owing to their high surface area, tunable porosity, and catalytic metal centers [127]. Details on MOF synthesis, structure, and application to polyolefin separators have already been covered in great detail in excellent reviews elsewhere [128,129,130]. Instead of modified polyolefin separators, we discuss exemplary investigations of modifying novel NF separators with various MOFs.

**Figure 8:**
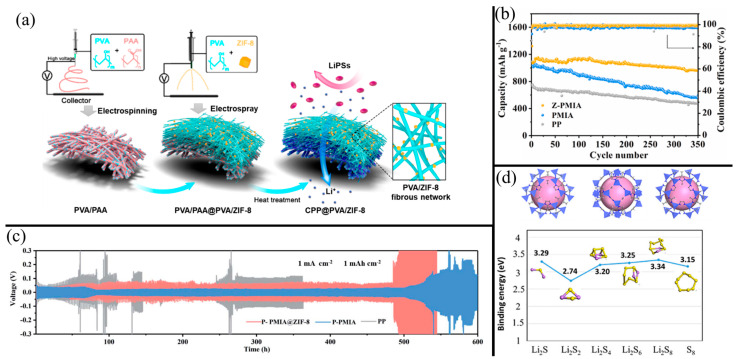
(**a**) Schematic diagram illustrating the fabrication of cross-linked PVA/PAA modified with ZIF-8 MOF. Reprinted/adapted with permission from Ref. [131]. (Copyright 2021, American Chemical Society). (**b**) The capacity retention of an LSB with a PP, PMIA NF, and ZIF-modified PMIA NF separators. Reprinted/adapted with permission from Ref. [73]. (Copyright 2021, Elsevier). (**c**) Li plating/stripping performance in symmetric Li cells with PP, PVDF-HFP/PMIA, and ZIF-8-modified PVDF-HFP/PMIA separators. Reprinted/adapted with permission from Ref. [21]. (Copyright 2022, Royal Society of Chemistry). (**d**) DFT skeletons of ZIF-67 surrounding a substrate from 3 orthogonal viewpoints and the binding energy of various LiPSs and S_8_ to the MOF as a substrate in the MOF cavity. Reprinted/adapted with permission from Ref. [117]. (Copyright 2020, Elsevier).

Figure 8a illustrates the fabrication process of a composite NF separator modified with ZIF-8 MOFs. Zheng et al. [131] first prepared the bottom layer by electrospinning a PVA/PAA solution. A PVA/ZIF-8 solution was then electrosprayed onto one side of the NF substrate at a high voltage of 30 kV. The composite NF was finally heat treated at 120 °C to promote the cross-linking of PVA and PAA and improve the anchoring of ZIF-8 on the polymer NFs. The composite NF (referred to as CPP@PVA/ZIF-8) improved electrolyte uptake due to the high surface area of the ZIF-8. However, the ZIF-8 slightly decreased the tensile strength and Li-ion conductivity compared to the unmodified cross-linked PVA/PAA NF separator but was still much greater than a standard PP separator. In contrast, a ZIF-modified PMIA separator had showed improved tensile strength and electrolyte affinity than an unmodified separator. In this study, Liu et al. [73] modified a PMIA NF separator with Co-containing zeolitic imidazolate framework (ZIF-L(Co)) via an in situ crystallization method. Thus, MOFs do not necessarily weaken the mechanical structure of NFs and may only slightly weaken the structure or may strengthen the NF separator instead. Regardless of the physical effects of adding MOFs, MOFs are known to trap LiPSs to suppress the shuttle effect. As shown in Figure 8b, the capacity of the PMIA separator with ZIF-L(Co) modifications had one of the smallest reported capacity decay rates of 0.03% per cycle over 350 cycles at 0.2 C. In contrast, the PMIA separator has a similar initial capacity that quickly decays, ending with a 51% retention. In addition to the trapping effect, the abundant metal centers in the MOFs have a high affinity for LiPS adsorption. Figure 8d shows the high binding energies of the various polysulfides to a ZIF-67 MOF. This is due to the Co-S, N-S, and O-S bonds made available by the porous MOFs [117]. Thus, the MOF-modified PAN/rGO-PAN separator also exhibited an extremely low-capacity fade rate of 0.03% per cycle after 600 cycles at 0.5 C. In a comparative study, LSBs with ZIF-67-modified ANFs have only slightly lower capacity retention than Fe_2_O_3_-modified ANFs and MoS_2_-modified ANFs [58]. This highlights the adsorptive affinity of ZIF-67 for LiPSs despite having a bulkier and less polar structure.

Adding MOFs to NF separators can regularize Li-ion deposition, reducing dendrite formation. Figure 8c shows the Li-ion stripping/plating performance of a symmetric Li cell with PP, PVDF-HFP-modified PMIA NF (P-PMIA), and PVDF-HFP/ZIF-8-modified PMIA NF (P-PMIA@ZIF-8) separators. Liu et al. [21] found that adding ZIF-8 greatly reduces the overpotential of the symmetric cell and helps limit polarization even at high areal capacities of 3.5 mAh cm^−2^. This was due to the ZIF-8 promoting homogeneous nucleation of Li and guiding Li-ions for uniform deposition. Li et al. [73] attributed the stable voltage profiles of Li stripping/plating largely to the uniform pore sizes of the MOF and the larger NF separator. Feng et al. [132] attributed the dendrite mitigation to minimum interface impedance due to the spider-web-like structure of ZIF-8-modified PVDF NFs.

Deng et al. [133] investigated the performance of an LSB with PMIA NF separators modified with two common MOFs: Co-based ZIF-67 and Cu-based HKUST-1. Adding the MOFs decreased the average pore size of the PMIA NFs, with HKUST-1-modified PMIA having around a 10% larger pore size than ZIF-67-modified NF and around a 50% smaller pore size than unmodified PMIA. The overall porosities of the MOF NFs were nearly identical and much greater than the PMIA and PP separators. Consequently, the electrolyte uptake was higher with MOFs, with HKUST-1 having slightly higher electrolyte wettability and uptake. Following this trend, the HKUST-1-modified had slightly higher ionic conductivity, lower charge transfer resistance, initial specific capacity, Coulombic efficiency, and capacity retention. The main differentiator between HKUST-1 and ZIF-67 was the higher rate capability and reversibility, likely due to the higher conductivity of the Cu metal centers. While more comparative studies should be conducted to suggest a “better” MOF, many MOFs will likely perform similarly when it comes to adsorbing and trapping LiPSs.

### 5.4. Alternative Composites

Inorganic nitrides such as TiN, ZrN, VN, and BN are promising materials for LSBs because of their high affinity for LiPSs, high electrical conductivity, and thermal stability [134]. Recently, Shi et al. [89] coated a polyvinylidene fluoride-polymethylmethacrylate (PVDF-PMMA) NF separator with VN on the cathode side and BN on the anode side via magnetron sputtering. The addition of VN and BN did not significantly affect the porosity and slightly increased electrolyte uptake due to increased electrolyte affinity with the inorganic nitrides. Similar to carbon nanoparticles (Section 5.1), the BN modifiers in the NF separator were able to uniformly and quickly dissipate heat, resulting in a higher melting temperature than the unmodified NF separator. Moreover, the modified separator can better resist fluctuations in temperature with higher thermal conductivity, which is critical for real-world applications. The high electrical conductivity of the nitrides reduced the charge transfer resistance by 28%, resulting in improved rate capability for the LSB. The VN particles on the sulfur cathode side showed excellent LiPS rejection via chemisorption that helped suppress self-discharge and a small 15% decay in specific capacity after 200 cycles.

Similar to transition metal oxides discussed in Section 5.1, SiO_2_ is excellent at adsorbing LiPSs to suppress the shuttle effect [135,136]. SiO_2_ is also abundant, affordable, and environmentally harmless, which makes it promising for industrial scale-up. Li et al. [85] modified oxidized bacterial cellulose NFs with SiO_2_ via in situ crystallization of SiO_2_ on the NFs. Compared to the unmodified BC NF separator, the SiO_2_-modified separator had slightly improved electrolyte wettability and Li-ion conductivity due to the abundant surface oxygen groups in the SiO_2_. The main advantage of adding SiO_2_ was the adsorption of LiPSs to suppress the shuttle effect. The LSB with a BC/SiO_2_ separator had better resistance against self-discharging and long-term capacity retention than with a BC separator. Xu et al. [108] modified PAN NFs with SiO_2_ nanoparticles via a one-step electrospinning process. The SiO_2_/PAN NF layer was placed facing the Li anode and was combined with a TiO_2_-modified CNF layer facing the cathode. Both the TiO_2_ and SiO_2_ could immobilize the LiPSs with strong binding energies, resulting in a high 75% capacity retention after 100 cycles at 0.2 C. In contrast, the capacity retentions of an LSB with PP and PAN NF separators were 56% and 53%, respectively.

Inorganic-organic hybrid materials like polyhedral oligomer silsesquioxanes nanoparticles have the advantages of high solubility with polymer solutions for facile electrospinning and high functionalizability for improved LiPS rejection. Zhao et al. [79] fabricated a PMIA NF separator with octaphenyl polyhedral oligomer silsesquioxanes (OAPS) nanoparticles via a facile electrospinning process. Fibers with higher OAPS content had smaller diameters due to the higher conductivity of the silicon in OAPS, affecting the charge density of the polymer solution. Increasing the OAPS content also increased the electrolyte affinity and uptake of the NFs, owing to the polar amino groups. The OAPS nanoparticles also restricted the movement of the PMIA NFs, resulting in improved resistance against thermal deformation. The highly electronegative amino groups also improved LiPS rejection via Coulombic repulsion, indicated by the 46% decrease in specific capacity after 800 cycles at 0.5 C. In contrast, the LSB with a polyethylene separator had a 75% decrease after only 500 cycles. Due to the highly customizable nature of polyhedral oligomer silsesquioxanes, there are many more untested yet promising functional groups that may further improve LSB cyclability.

Biologically derived modifiers that have high binding energies with LiPSs are especially promising because such modifiers open up the possibility of recycling waste materials. This is especially important for batteries that must be replaced regularly due to decaying capacity. Yang et al. [80] modified PMIA NFs with starch, which is one of the most common carbohydrates found abundantly in essential crops like wheat, potatoes, and rice. Increasing starch content increased electrolyte wettability and uptake due to the many –OH and C-O-C groups in starch that have a high affinity for the electrolyte. These polar groups also helped adsorb the LiPSs. The pore sizes also decreased with the addition of starch, improving LiPS rejection via physical sieving. Thus, the shuttle effect was greatly mitigated, with a 0.9% decay per cycle with starch compared to a 1.3% decay per cycle without starch. Chen et al. [90] combined electrospun CNFs with electrospun gelatin proteins to form a bilayer Janus-type separator. The protein-based layer was on the Li anode side because it was the necessary insulating layer to prevent short-circuiting in the LSB. The conductive CNF layer was also coated with the gelatin proteins via drop casting. The amine and carboxyl groups in the gelatin improved electrolyte affinity and ionic conductivity, which decreased Li dendrite formation. The polar groups also have strong interactions with LiPSs, and the complex structure of the proteins helps trap the polysulfides to suppress the shuttle effect.

**Table 2:** The performance and notable developments of LSBs with various modifiers in order of carbon, metal oxide, MOF, and alternative modifiers.

Polymer Material	Modifier	Initial Capacity (mAh g^−1^)	Current Density (C)	# of Cycles	Capacity Decay (% per Cycle)	Highlights	Ref.
PAN/PVDF-HFP	CB/VOOH	811	2	500	0.16	Cross-linking even with carbon embedded in the NFs yielded more mechanically robust and thermally stable NFs	[52]
PVDF	rGO	1322	0.2	200	0.26	rGO improved rate capability by decreasing charge transfer resistance and improving redox kinetics	[88]
PAN	GO	987	0.2	100	0.4	GO provided electrostatic repulsion against anionic polysulfides	[116]
CNF/PAN	CeO_2_	1001	0.5	300	0.04	CeO_2_ acted as an electrocatalyst that improved LiPS reduction from long to short chains	[45]
PAN	Al_2_O_3_	947	0.2*	100	0.68	Metal sites on Al_2_O_3_ improved NF affinity for electrolyte	[53]
ANF	Fe_2_O_3_	1080	1	1000	0.24	Fe_2_O_3_ increased NF affinity for LiPS adsorption	[58]
PAN	CTP & LLZTO	1288	0.5	500	0.06	LLZTO provided conductive pathways for fast and uniform Li ion diffusion and deposition	[126]
PMIA/PVDF-HFP	ZIF-8	1156	0.2	300	0.09	ZIF-8 promoted the uniform nucleation and deposition of Li via uniform pore structures	[21]
PMIA	ZIF-L(Co)	1391	0.2	350	0.03	MOF modification improved tensile and puncture strength P	[73]
PAN	ZIF-67 & rGO	485	5	600	0.03	ZIF-67 possessed multiple binding sites for excellent LiPS adsorption	[117]
PVA/PAA	ZIF-8	1125	0.1	300	0.05	The ZIF-8 improved rate capability due to improved redox kinetics with ZIF-8 metal centers	[131]
PVDF	ZIF-8 & TBAC	1324	2	700	0.05	ZIF-8 interfered with PVDF crystallinity, resulting in a more amorphous structure that decreased interface impedance with the anode	[132]
PMIA	HKUST-1	1272	0.5	500	0.08	The slightly higher conductivity of Cu than Co likely the rate capability of HKUST-1 over ZIF-67	[133]
PMIA	OAPS	851	0.5	800	0.06	OAPS improved LiPS rejection via Coulombic repulsion	[79]
PMIA	starch	1118	0.5	500	0.90	Polar groups on the starch improved electrolyte affinity	[80]
BC	SiO_2_	1250	0.25	100	0.17	SiO_2_ adsorption of LiPS improved cycle stability	[85]
PVDF/PMMA	VN & BN	1077	0.2	200	0.1	Thermally conductive BN distributed heat uniformly for fast dissipation and higher thermal stability	[89]
PVP CNF	gelatin	890	0.5 *	300	0.12	Gelatin coating endowed CNFs with polar groups with affinity for LiPS	[90]
CNF/ PAN	SiO_2_ & TiO_2_	996	1	1000	0.06	SiO_2_ on the Li anode side contributed to LiPS adsorption for restricted shuttling	[108]

* Current density measured in A g^−1^ instead of C rate. PAN (polyacrylonitrile); PVDF-HFP (poly(vinylidene fluoride-co-hexafluoropropylene)); CB (carbon black); ANF (aramid nanofiber); CTP (covalent triazine piperazine); LLZTO (Li_6.4_La_3_Zr_1.4_Ta_0.6_O_12_); ZIF-L(Co) (Co-containing zeolitic imidazolate framework); PVDF-HFP (poly(vinylidene fluoride-co-hexafluoropropylene)); TBAC (tetrabutylammonium chloride); BC (bacterial cellulose); OAPS (octaphenyl polyhedral oligomer silsesquioxanes); PVP (polyvinylpyrrolidone).

## 6. Future Perspectives

NF separators are superior alternatives compared to commercially available polyolefin separators for LSBs because of their greater thermal stability, ionic conductivity, electrolyte affinity, Li dendrite-suppressing capability, and LiPS-rejecting ability [25,26]. Most of the latest research has gone into modifying polyolefin separators with interlayers or other modifiers [137,138,139,140,141,142,143] to ease the transition from Li-ion to LSBs at the industrial scale. However, there are clear limits to thermal stability, ionic conductivity, and LiPS suppression for LSBs if polyolefin separators remain the base separator. Similarly, many new materials are tested as cathode materials for their excellent LiPS affinity, thermal stability, and electrolyte affinity that have potential as modifiers for NF separators [144,145,146,147,148,149]. There is untapped potential in NF separators, especially if we use the various materials and lessons learned from modifying polyolefin-based separators and cathode materials.

MXenes are an example of newer materials that have gained popularity as modifiers for polyolefin separators and LSB cathodes. Their popularity is due to their high conductivity, tunable functional groups, hydrophilicity, and strong attraction for LiPSs [17]. However, we are unaware of any publications for MXene-modified novel NF separators. Similarly, covalent organic frameworks (COFs) have been investigated as modifiers for various LSB components because of their tunable porosity, strong interaction with LiPS, and various functional groups [150]. However, we are only aware of one study by Wu et al. who modified a PAN NF separator with covalent triazine piperazine [126]. Other promising materials like catalytic chalcogenides [151], layered double hydroxides [152], quantum dots [153], and metal borides [154] have yet to be investigated as modifiers for NF separators.

While there have already been various publications on carbon, metal oxide, and MOF composite NFs, there are many materials within those broad categories that have only sparsely or have yet to be applied to NF separators. For example, ZIF-8 and ZIF-67 are commonly tested MOFs as discussed in Section 5.3, but there are many other MOFs, such as MOF-808 [155], MIL-100 [156], and UiO_66_ [157], that have not been applied to NF separators. Similarly, there are more metal oxides than those examined in Section 5.2 and carbon nanomaterials examined in Section 5.2 that may suppress the shuttle effect and improve LSB performance.

Many of the studies analyzed in this review have also not done any optimizations for the fabrication process of the NFs. Because of the facile setup and tuning of electrospinning, various optimization studies may be required for a better understanding of what pore sizes, fiber thicknesses, and polymer functional groups make exceptional NF separators for LSBs. Moreover, other nanofiber-making methods, including needle-less electrospinning, melt processes, or other commercial-scale spinning methods, may be investigated more thoroughly to test the viability of promising separators in the real world.

## 7. Conclusions

NF separators are promising alternatives to polyolefin-based separators because of their naturally higher thermal stability, porosity, Li dendrite-suppression capability, and LiPS-rejection ability. Various polymers such as PAN, PI, PVA, PVDF, cellulose, and aromatic polyamides can be easily and quickly turned into NFs via electrospinning, which can be fine-tuned for controllable porosity, density, and thickness by controlling simple parameters such as the solvent, feed rate, and operating voltage. These NFs can physically trap LiPS and have functional groups with strong interactions that immobilize LiPSs. Polymers with polar groups, such as PI, PVA, aramid, and cellulose, exhibit the ability to immobilize LiPSs. Moreover, polymers can be doped with F or functionalized with electronegative groups to have stronger interactions with LiPSs and electrolyte affinity. Cross-linking shrinks pore sizes while maintaining high porosity, enabling better LiPS rejection via ionic sieving. The tighter pore structure also improves thermal stability and mechanical strength. Polymer NFs can also be turned into highly conductive CNFs through a facile carbonization step to improve the rate capability and capacity retention of LSBs. Conductive CNFs improve the redox kinetics as LiPS due to higher electron mobility, reducing soluble LiPS into insoluble LiPS to prevent polysulfide build-up and diffusion. Despite suppressing the shuttle effect better than polyolefin separators, NF separators often require modifications to improve LSB cyclability. Promising additives are conductive, polar, and catalytic, which promotes the repulsion, trapping, chemisorption, and redox of LiPS. Common additives include conductive carbon-based nanoparticles such as graphene oxide that improve the rate capability and LiPS conversion. Catalytic metal oxides such as TiO_2_ increase sulfur utilization by enhancing the redox kinetics of the LSB. Porous MOFs such as ZIF-8 have a high affinity for LiPSs and can easily trap soluble LiPSs with its highly porous structure. Other promising materials exhibit some mixture of high conductivity, strong LiPS interactions, and good catalytic activity. Biologically derived additives are also promising for their sustainable development. Thermally conductive materials help dissipate excess thermal heat and distribute the heat uniformly to suppress thermal shrinkage.

## Data Availability

Not applicable.
